# Application of inter‐professional care model in patients with aneurysmal subarachnoid haemorrhage

**DOI:** 10.1111/jonm.12993

**Published:** 2020-04-20

**Authors:** Juan Xu, Jun Wu, Haihua Yan

**Affiliations:** ^1^ Shanxi People's Hospital Taiyuan China; ^2^ Graduate School of Shanxi University of Traditional Chinese Medicine Taiyuan China

**Keywords:** aneurysmal subarachnoid haemorrhage, conventional holistic care, inter‐professional care model, nursing

## Abstract

**Objective:**

To explore the feasibility and effect of the inter‐professional care model in patients with aneurysmal subarachnoid haemorrhage.

**Methods:**

A convenient sampling method was used to recruit inpatients of a hospital as subjects from July 2016 to July 2018. According to the even/odd attribute of admission number, subjects were divided into a control group and an observation group. The number of recruited subjects was 311: the control group comprised 135 participants and the observation group 176. The average length of hospital stay, hospital fees, quality of life, and satisfaction with the quality of nursing were compared between the two groups. SPIRIT checklist was completed (see File S1).

**Results:**

After intervention, patients in the observation group had shorter average hospital stay (15.98 ± 2.7), lower hospital fees (81,018 ± 1.3), higher satisfaction with the quality of nursing (98.3%), lower incidence of complications (19.89%), improved ability to perform activities of daily living, and lower rate of disease outcome and re‐admission, with statistically significant differences from the control group (*p* < .05).

**Conclusion:**

The application of inter‐professional care model in single disease patients with aneurysmal subarachnoid haemorrhage can shorten the average hospital stay, reduce hospital fees, improve the quality of life of patients, and increase patients’ satisfaction with the quality of nursing, which is worthy of clinical promotion and application.

**Implications for nursing management section:**

Nursing managers can use this model to improve the ability to ensure coordination between medical professionals and integrate the ability of nursing problems, the ability to make rational distribution of nursing human resources, and the ability of critical thinking. It can be used as reference to improve the nursing management of all kinds of single diseases.

## 1 INTRODUCTION

Intracranial aneurysmal subarachnoid haemorrhage (aSAH) is an acute cerebral vascular disease that severely damages the central nervous system and has pathological effects on multiple organs in the body. Wang (2015) and Xu, Wang, Hu, and Liang (2015) stated that the aSAH patient has rapid onset, critical symptoms, volatile conditions, difficult nursing and 50% mortality rate after onset within 1 year (Kiiski et al., 2018), which suggests higher demand for nursing.

## 2 BACKGROUND

Xu, Li, Pei, An, and Wu (2019) conducted an investigation and a study on the status quo of the mental resilience of aSAH patients in the early stage, and the results showed that the overall level of the mental resilience of aSAH patients was relatively low. It was found that a stronger communication between nurses and patients could help improve the level of mental resilience, which was considered a reference for subsequent studies. The conventional care model is solely implemented by nurses, with limited nursing measures, relatively low level of medical cooperation, long hospital stay and high treatment fees, which increases the burden on patients and their families. In recent years, the model of ‘integrated medical care’ has been applied to many disciplines such as orthopedics, neurology and neurosurgery. Ying, Yang, and Liu (2019) selected 401 elderly patients with intertrochanteric fractures for a clinical randomized controlled trial, and the observation group formed an integrated medical care group. After training, the clinical care path management was implemented in a multidisciplinary joint manner. The results showed that the integration of care effectively increased patient satisfaction by 15.27%, compared with the control group, and effectively reduced delirium incidence by 29.96% and postoperative 6‐month mortality by 29.04%. Pang, Zhan, and Chen (2018) chose to apply the ‘medical integration’ model in 24 patients with acute cerebral infarction during their hospitalization and conducted telephone follow‐up one and three months after intravenous thrombolysis. The results showed that the quality of life of the patients in the observation group was significantly higher than that in the control group. Xu, He, and Zhao, He and Zhao (2017) showed that the inter‐professional care model can shorten the average length of stay of patients, reduce the rate of complications and improve the satisfaction with the quality of nursing.

## 3 METHODS

General information: from July 2016 to July 2018, a total of 311 inpatients with aneurysmal subarachnoid haemorrhage in one public hospital were recruited as subjects according to convenient inclusion criteria: (1) patients having aneurysmal subarachnoid haemorrhage after 1 week; (2) patients between 20 and 70 years old; and (3) patients with Hunt classification of grade 0 ~ IV. Exclusion criteria were as follows: (1) patients with both serious cardiovascular and cerebrovascular diseases; (2) patients having recent head trauma or pseudoaneurysm; and (3) patients having concurrent diseases other than brain diseases. Eligible patients were divided into groups according to the odd/even mantissa of the inpatient admission number. Those with odd mantissa were assigned to the control group, and those with even mantissa were assigned to the observation group.

Data collection methods: researchers, trained graduate nursing students and clinical nurses formed a survey team, and an on‐site questionnaire to guide patients to fill in the questionnaire. Those who could not fill in the questionnaire were asked by the investigators to fill it in item by item. The nursing quality satisfaction questionnaire was completed by the patient or a long‐term accompanying family member on the day of discharge.

Standard Protocol Items: ‘Recommendations for Interventional Trials’ (SPIRIT) checklist was completed (see File S1).

This study met the requirements of the Helsinki Declaration formulated by the World Health Organization. Study subjects gave written informed consent.

Intervention design: the control group received conventional holistic nursing, which assessed the existing health problems at different stages of the disease course and provided corresponding nursing measures. Nurses make judgements based on doctors' prescription and accordingly develop care plans for patients. The conventional holistic care model is solely run by nurses.

The integrated nursing model of care was applied to the observation group as follows: the inter‐professional care team: 36 experienced nurses were selected first.

According to professional titles, there were 4 nurses, 24 advanced practice nurses, 4 nurse supervisors and 4 deputy chief nurses. According to the random number function of Excel to generate randomization sequences, 36 nurses were divided into two groups, and there was no difference in age and hierarchy ability between the two groups (*p* > .05). One team of 18 nurses was assigned to the control group, and the other was assigned to the observation group. Apart from the conventional holistic nursing team, 2 rehabilitation therapists, 2 dietitians and 3 acupuncturists with 5 or more years of clinical work experience, the doctors specialized in cerebrovascular diseases were invited to join one of the nursing teams (18 nurses), and eventually to form the inter‐professional care team.

The implementation of inter‐professional care model: inter‐professional care refers to a clinical team formed by doctors, nurses, rehabilitation therapists and dietitians to provide comprehensive and holistic treatment and nursing for inpatients (in the same ward) with aneurysmal subarachnoid haemorrhage. Specific intervention methods were as follows. (1) Inter‐professional joint care team with early morning rounds: during the intervention, the medical team conducted rounds for patients under their care at 08:00 every day. Firstly, the patient stated his/her own concerns, the nurse reported the patient's vital signs and major nursing issues, and the doctor reported the patient's basic conditions. Secondly, based on the above information, the supervising physician and head nurse further conducted a comprehensive evaluation of the patient. (2) Post‐rounds meeting: according to the main issues of patients, nurses and doctors and the updated assessment from the rounds, the supervising physicians, head nurses, rehabilitation therapists and dietitians formulated the corresponding nursing plans and measures. (3) Implementation of nursing plans: based on the standard nursing plan developed by the hospital, the nurse revised the nursing plan of the respective patients and carried out specific measures. (4) Late rounds conducted by the inter‐professional care team: every day at 5:00 p.m., the team conducted clinical rounds for patients, focusing on the evaluation of nursing outcomes on each day, with attention given to the difficulties and unresolved nursing problems during the implementation of nursing process.

Evaluation indexes were as follows. (1) Average hospital stay: the average number of days from hospital admission to hospital discharge was compared, (2) The second evaluation index was hospital fees, (3) Nursing quality satisfaction: a self‐developed questionnaire was used, and the evaluation was divided into five grades: very satisfied, relatively satisfied, generally satisfied, dissatisfied and very dissatisfied. The satisfaction rate = very satisfied + relatively satisfied + generally satisfied cases/total satisfied cases × 100%. (4) Incidence of complications: the incidence of hypostatic pneumonia, or deep venous thrombosis, or pressure ulcers in lower limbs during hospitalization in the two groups was observed. (5) Activities of daily living (ADL): the Barthel index was used for evaluation, with the full score of 100 and ≤40 classified as heavily dependent, all requiring others' care; 41–60 classified as moderately dependent, most in need of care; 61–99 classified as mildly dependent with a few requiring others' care; and 100 classified as = no need for care. The ability to perform activities of daily living (ADL) of the observation group was better than that of the control group, with statistical significance at *p* < .001 (see Table 2). (6) Disease return and re‐admission rate: the outcomes of disease return were cure, improvement, death and others.

Statistical methods: SPSS 25.0 software was used for statistical analysis. Frequency and rate (%) were used for descriptive statistics. Chi‐square test and rank sum test were used for comparison. The measurement data were expressed as mean ± standard deviation ( $\bar{x}\pm s$ ), and *t* test was used for comparison. Statistical significance was set at *p* < .05.

## 4 RESULT

In this study, a total of 315 questionnaires were issued and 311 valid questionnaires were collected, with an effective recovery rate of 98.7%, which met the requirements of the investigation. From July 2016 to July 2018, a total of 311 inpatients with aneurysmal subarachnoid haemorrhage in one public hospital were recruited as subjects: 134 were male and 177 were female, with an average age of 54 years. There were 135 cases in the control group, including 62 males and 73 females with the following characteristics: age (53.7 ± 2.4 years); Hunt classification: 8 patients (Grade 0), 85 patients (Grade I), 23 patients (Grade II), 13 patients (Grade III), 5 patients (Grade IV) and 1 patient (Grade V). There were 176 cases in the observation group, including 72 males and 104 females with the following characteristics: age (54.8 ± 3.1 years); Hunt classification: 9 patients (Grade 0), 99 patients (Grade I), 28 patients (Grade II), 26 patients (Grade III), 14 patients (Grade IV) and 0 patients (Grade V). There were no statistically significant differences in gender, age, Hunt classification or other general information between the two groups (*p* > .05), suggesting comparability.

**FIGURE 1:**
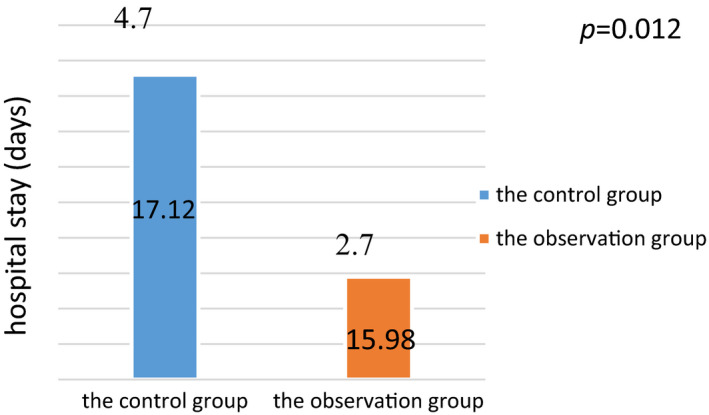
Comparison of hospitalization duration between the two groups [Colour figure can be viewed at wileyonlinelibrary.com]

**FIGURE 2:**
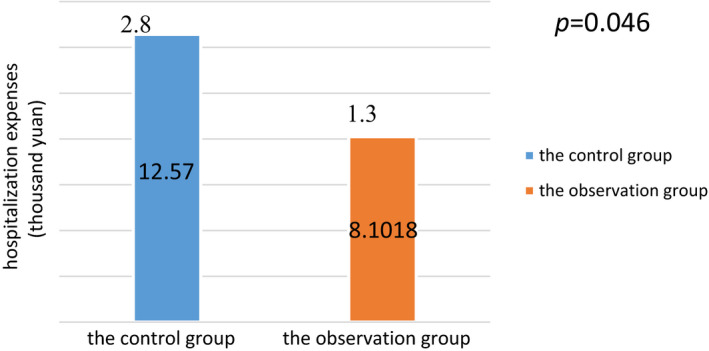
Comparison of hospitalization expenses between the two groups [Colour figure can be viewed at wileyonlinelibrary.com]

Comparison of hospitalization duration and hospitalization fees between the two groups: after intervention, the average length of stay in the observation group was 15.98 ± 2.7 days, while that of the control group was 17.12 ± 4.7 days, with statistical significance at *p* = .046 (see Figure 1). Hospital fees in the observation group were 8.10 ± 1.3 thousand RMB, while those in the control group were 12.57 ± 2.8 thousand RMB, with statistical significance at *p* = .046 (see Figure 2).

Comparison of nursing quality satisfaction between the two groups: after intervention, the satisfaction with nursing quality in the observation group (98.3%) was higher than that in the control group (94%), with statistical significance at *p* = .020 (see Table 1).

**TABLE 1:** Comparison of nursing quality satisfaction between the two groups (%)

Group	*n*	VS *n* (%)	S *n* (%)	NS *n* (%)	Rate (%)	χ^2^	***p***
Control	135	58 (43)	69 (50)	8 (5)	94.0	11.638	.020
Observation	176	104 (59)	69 (41)	3 (1)	98.3		

Abbreviations: S, Satisfactory, and NS, Not Satisfactory; VS, Very Satisfactory.

**FIGURE 3:**
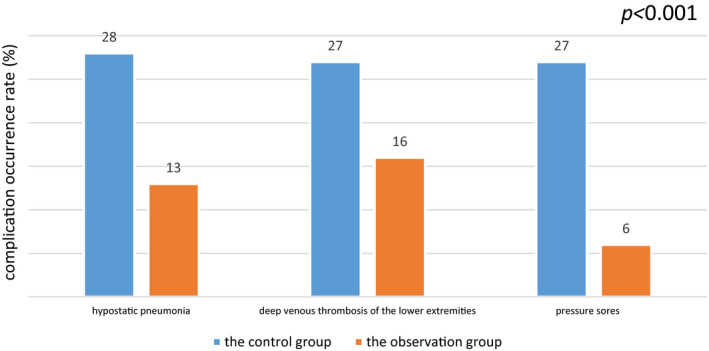
Comparison of occurrence rates of complications between the two groups (%) [Colour figure can be viewed at wileyonlinelibrary.com]

Comparison of complications: after intervention, the percentage occurrence rate of hypostatic pneumonia in the observation group was 13%, while that of the control group was 28%, with statistical significance at *p = *.001. The percentage occurrence rate of deep venous thrombosis in the observation group was 16%, while that of the control group was 27%, with statistical significance at *p* = .019. The percentage occurrence rate of pressure ulcers in the observation group was 6%, while that of the control group was 27%, with statistical significance at *p* ***= ***.000 (see Figure 3).

Comparison of the ability to perform activities of daily living between the two groups: after intervention, the percentage occurrence rate of the ability to perform activities of daily living (ADL) score was less than or equal to 40 in the observation group, which was 9% as compared to that of the control group 21%. The percentage occurrence rate of the ADL score (41–60) in the observation group was 32%, while that of the control group was 44%. The percentage occurrence rate of the ADL score (61–99) in the observation group was 56%, while that of the control group was 34%. The percentage occurrence rate of the ADL score (100) in the observation group was 56%, while that of the control group was 34%. The ability to perform activities of daily living (ADL) of the observation group was better than that of the control group, with statistical significance at *p < *.001 (see Table 2).

**TABLE 2:** Comparison of the ability to perform activities of daily living between the two groups (%)

Group	*N*	Barthel index score (0–100)				Z	*p*
		≤40 *n* (%)	41–60 *n* (%)	61–99 *n* (%)	100 *n* (%)		
Control	135	28 (21)	59 (44)	46 (34)	2 (1)	4.351	<.001
Observation	176	16 (9)	56 (32)	98 (56)	6 (3)		

Note

Barthel index score: ≤40, heavily dependent; 41–60 moderately dependent; 61–99, mildly dependent; and 100, independent.

Disease outcome and re‐admission in both groups: after intervention, the percentage occurrence rate of the cure in the observation group was 36%, while that of the control group was 27%. The percentage occurrence rate of taking a cure was better in the observation group (61%) than that in the control group (63%). The percentage occurrence rate of death in the observation group was 1%, while that of the control group was 4%. The percentage occurrence rate control in the observation group was 2%, while that of the control group was 6%. The disease outcome of the observation group was higher than that of the control group, with statistical significance at *p = *.011 (see Table 3).

**TABLE 3:** Comparison of disease outcomes between the two groups (%)

Group	*N*	Prognosis of disease				χ^2^	*p*
		Cure	Improvement	Death	Others		
Control	135	36 (27)	85 (63)	6 (4)	8 (6)	11.215	.011
Observation	176	64 (36)	108 (61)	1 (1)	3 (2)		

**FIGURE 4:**
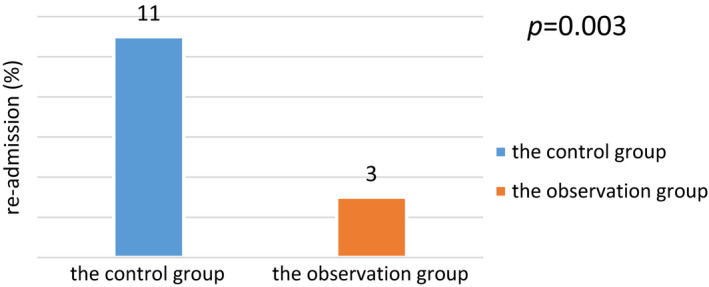
Comparison of re‐admission between the two groups (%) [Colour figure can be viewed at wileyonlinelibrary.com]

The percentage occurrence rate of re‐admission in the observation group was 3%, while that of the control group was 11%. The re‐admission of the observation group was lower than that of the control group, with statistical significance at *p* = .003 (see Figure 4).

## 5 DISCUSSION

Inter‐professional care model is a new clinical care model. Chen, Zhang, and Xie (2011); Chao, Wang, and Zhao (2018); and Zhong, Liu, and Yang (2019) suggested that the inter‐professional care model should involve a team of health care professionals to carry out a comprehensive assessment of a certain disease, set nursing diagnosis, propose nursing problems, and record on the nursing plans during the hospitalization of patients. This model is applicable to specific groups of patients with the control group was 27% single disease.

The authors defined a single disease patient as a patient suffering from a disease or disease‐related complications. The subjects of this study were patients with single species of aneurysmal subarachnoid haemorrhage. This model is comprehensive, with the characteristic of integrating together evidence‐based medicine, holistic nursing, clinical pathway and continuous quality improvement.

The effect of inter‐professional care is seen in shortening the average hospital stay of patients and in reducing hospital fees. As per Figures 1 and 2, it is shown that the average length of stay in the observation group was 2 days, which is shorter than that in the control group, and the hospitalization cost was 44,682 yuan, which is lower than that in the control group. The results of this study are consistent with those of the research of Xu et al. in, 2017. On the one hand, in the conventional model, nurses implemented nursing care on the orders of doctors.

However, they were less involved in the treatment plan of individual patients. They slightly considered the impact of nursing service on the significance of the patient rehabilitation. Furthermore, the communication between patients, doctors and nurses was fragmented. It makes communication difficult and ignores the patient's real problems. On the other hand, the inter‐professional care model allowed better communication between doctors and nurses about the operation process, the course of disease, and the treatment principles, which is conducive to better nursing measures and outcomes. The face‐to‐face patient and team communication enhance mutual exchange and patient's adequate expression of their needs and concerns, resulting in timely attention and solutions. The patient can have a harmonious working relationship and a better treatment satisfaction and can experience reduction in clinical conflicts and increase in the satisfaction with the quality of nursing. In addition, better cooperation among the team of health care professionals and timely communication can improve the quality of care through revision of the holistic care plan, with specific reference to the patient's course of disease and his/her psychosocial conditions.

The inter‐profession model improves patients' satisfaction with the quality of nursing. The satisfaction of patients in the observation group was 4.3%, which is higher than that in the control group (see Table 1). This was consistent with the research results of Zhang and Zhou (2016). On the one hand, in the conventional nursing model, the nursing staff carefully completed various nursing operations according to the doctor's advice and provided high‐quality nursing services, but the nursing staff were less involved in the diagnosis and treatment of patients' diseases and consideration of the significance of nursing services for patients' rehabilitation. This joint operation model of care can enhance the communication between health care professionals. Through doctors' explanation of patients' operation processes, disease progress and diagnosis, and treatment principles, the nursing staff can make corresponding nursing plans to better improve nursing measures and improve the nursing effect. On the other hand, the mode of communication between patients, doctors and nursing staff in the conventional nursing model is fragmented, and patients' needs are re‐iterated many times, which often leads to mutual excuses. The joint approach instead creates face‐to‐face opportunities for patients and the related medical and health care staff, enabling bi‐directional communication, well‐expressed mutual concerns, and immediate attention to the needs of patients. Patients themselves feel comfortable with the nurse–patient relationship (National Health Commission of the People's Republic of China, 2017), thus improving patients' treatment adherence and increasing nursing quality satisfaction. In addition, doctors, nurses and other relevant health care personnel cooperate closely with each other, communicate in a timely manner, adjust the nursing plan according to the patient's conditions and psychological dynamics, and improve the quality of nursing.

As per Figures 3, 4 and Tables 2, 3, it is shown that the observation group had lower incidence of postoperative complications, higher ability to perform activities of daily living, and better disease outcomes than those of the control group (*p* < .05). In the conventional nursing model, there are not many restrictions on the choice of nurses, and low‐level nurses can also take care of patients with severe diseases. However, aSAH patients suffer from sudden onset, critical symptoms and volatile conditions, which lay down the requirements for the selection of nursing staff. The selection of nurses in this study is based on the hierarchical management of nurses (Gan, Zhao, Xie, Xiao, & Kang, 2015). The more critical the patient is, the higher the level of nursing staff required.

Reasonable stratification of personnel at all levels can ensure scientific handling of nurses of nurses, improve the quality and efficiency of nursing (Zhao & Li, 2019), and thus improve the quality of life of patients. Medical and nursing models in the medical ward round and case discussion make the team communication more effective and efficient, accurate information comprehensive, plan adjustment timely, and observation points clear, and they provide detailed effective nursing measures. Moreover, they provide patients with predictive diagnosis and treatment and care services, reduce the complications of cases, and improve patients’ quality of life. The inter‐professional model of care strengthens the cooperation and communication between relevant disciplines and personnel, improves the adherence of patients, provides educational opportunities for relevant personnel, and better complements the service gap and utilizes the individual strengths of respective health care experts (Wu, Ma, & Liao, Ma & Liao, 2017).

## 6 CONCLUSION

The implementation of the inter‐professional model of patient care can shorten the average length of stay, reduce the cost of hospitalization/hospital fees, improve the quality of life of patients, and improve patients’ satisfaction with the quality of care, which is worthy of clinical promotion. In this study, we only applied the inter‐professional model of care to intracranial aneurysmal subarachnoid haemorrhage and other diseases. In the future, we need to conduct in‐depth research on the construction of standardized operational models of inter‐professional patient care and related service project indicators.

## 7 IMPLICATIONS FOR NURSING MANAGEMENT SECTION

Multidisciplinary and inter‐professional cooperation nursing management models, through the comprehensive assessment of the overall situation of patients by multidisciplinary medical staff, discuss nursing problems and put forward solutions and implement them, so that patients in a short space of time receive the best quality of care. Nursing managers can use this model to improve the ability to ensure coordination among medical professionals and integrate the ability of nursing problems, the ability to make rational distribution of nursing human resources, and the ability of critical thinking. It can be used as a reference to improve the nursing management of all kinds of single diseases.

## AUTHOR CONTRIBUTION

Xu Juan contributed to conceptual framework and design, paper revision and proof‐reading. Xu Juan, Wu Jun and Yan Haihua contributed to the implementation, data collection and analysis, and writing of the paper.

## ETHICAL APPROVAL

This study met the requirements of the Helsinki Declaration formulated by the World Health Organization. Participants gave written informed consent. Application of inter‐professional care model in patients with aneurysmal subarachnoid haemorrhage

## Supporting information

File S1
